# Supplementary material for: Exposure to Low Zearalenone Doses and Changes in the Homeostasis and Concentrations of Endogenous Hormones in Selected Steroid-Sensitive Tissues in Pre-Pubertal Gilts
Source: Toxins (Basel). 2022 Nov 11;14(11):790. doi: 10.3390/toxins14110790 (PMC9692984; doi:10.3390/toxins14110790)
Supplement: Supplementary file 1 [file toxins-14-00790-s001.zip › toxins-2012151-supplementary.pdf]

toxins

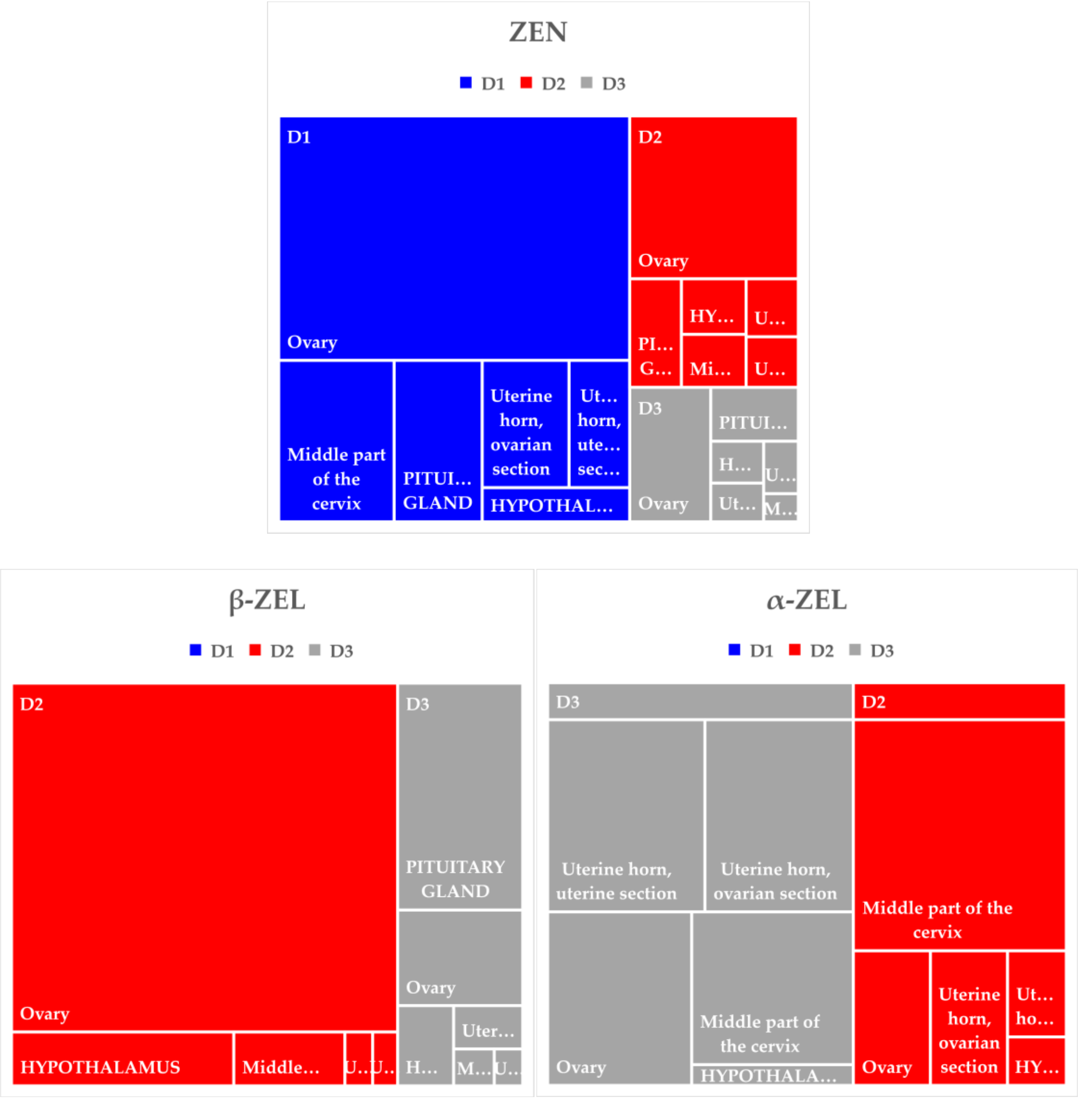

Figure S1. Tree map of ZEN, α-ZEL, and β-ZEL concentrations in reproductive system, hypothalamic, and pituitary gland tissues of Group ZEN5 gilts (5 μg ZEN/kg BW). Key: D1—exposure day 7; D2—exposure day 21; D3—exposure day 42.

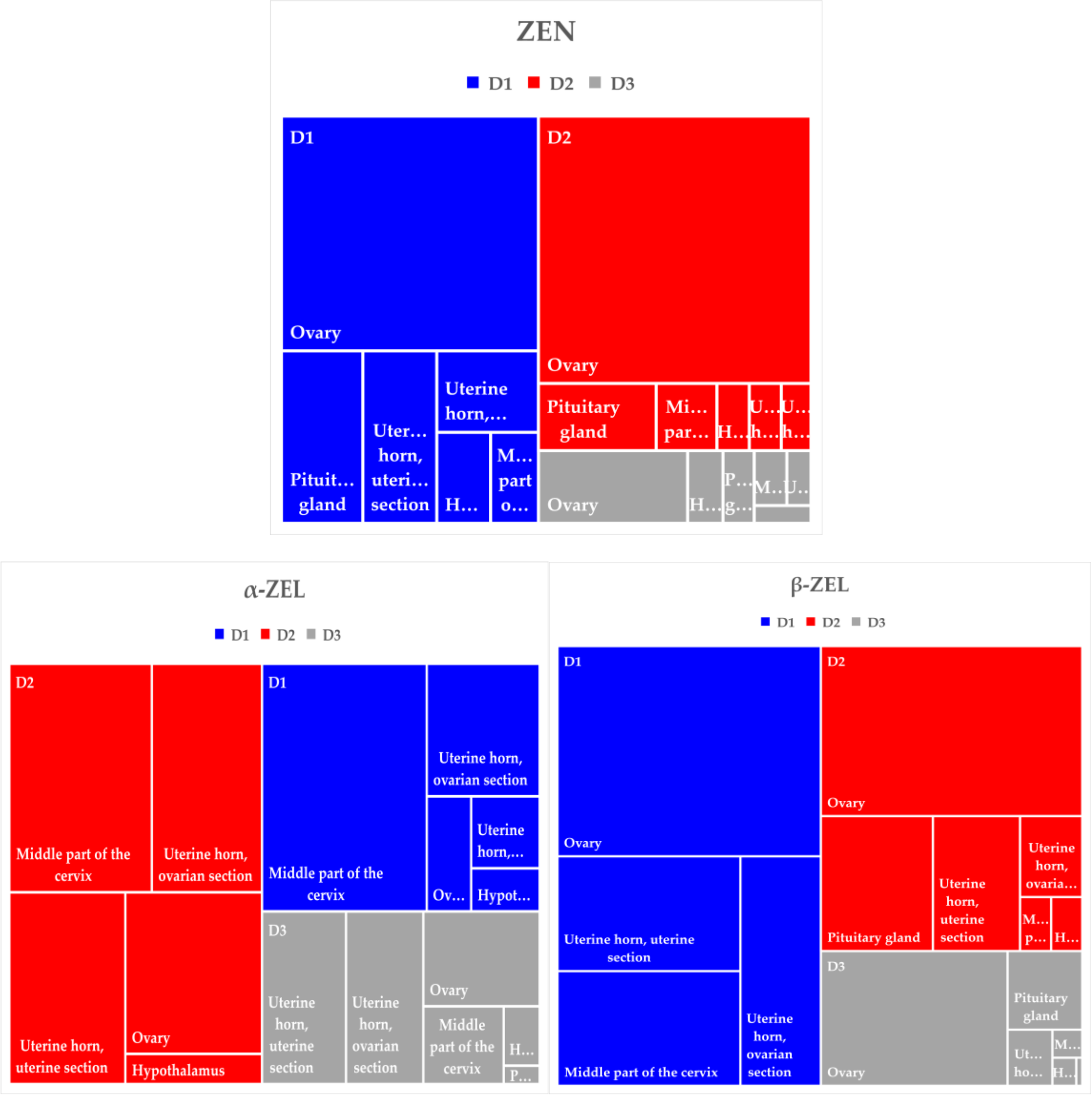

Figure S2. Tree map of ZEN, α-ZEL, and β-ZEL concentrations in reproductive system, hypothalamic, and pituitary gland tissues of Group ZEN10 gilts (10 µg ZEN/kg BW). Key: D1—exposure day 7; D2—exposure day 21; D3—exposure day 42.

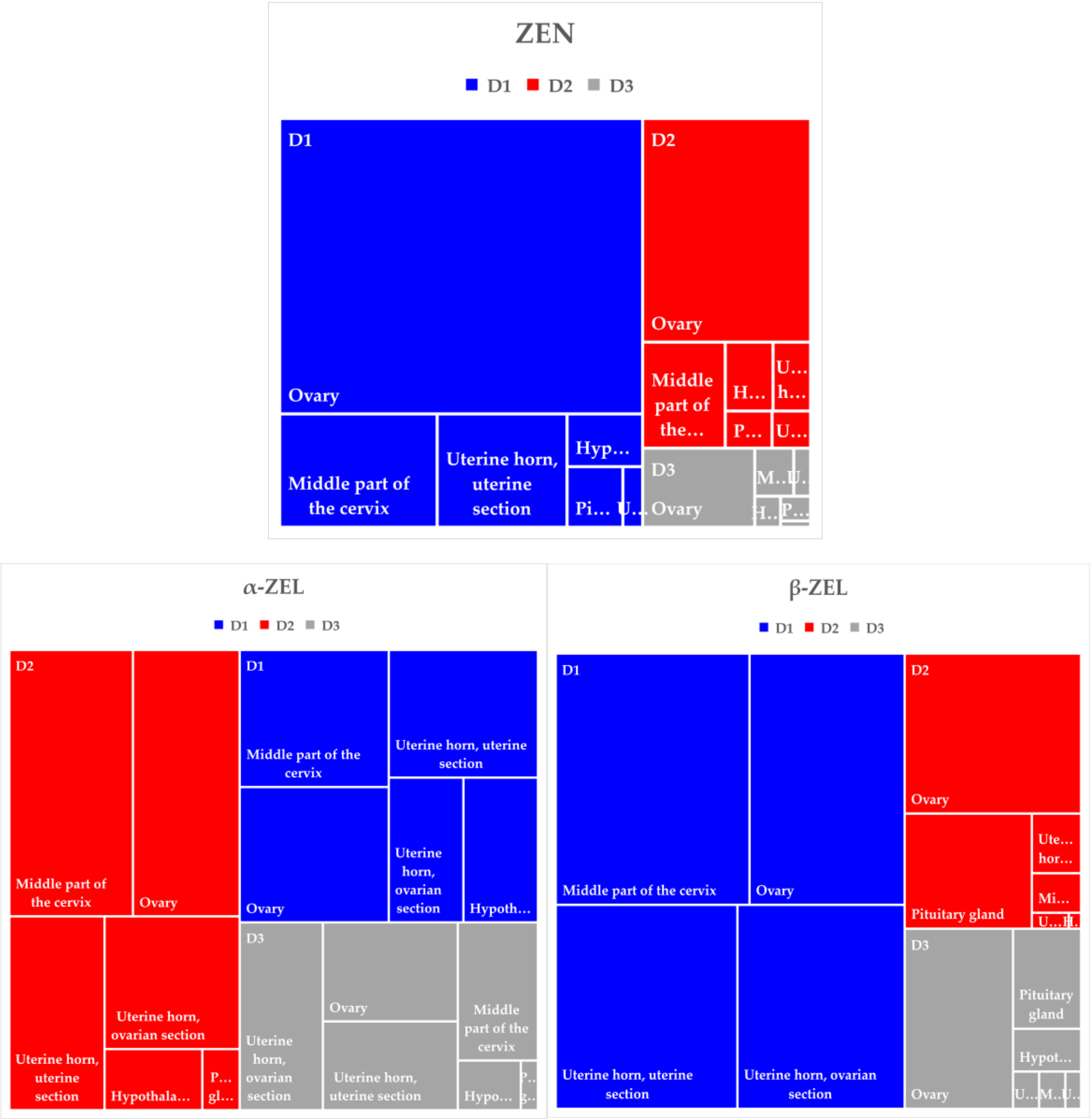

Figure S3. Tree map of ZEN, α-ZEL, and β-ZEL concentrations in reproductive system, hypothalamic, and pituitary gland tissues of Group ZEN15 gilts (15 µg ZEN/kg BW). Key: D1—exposure day 7; D2—exposure day 21; D3—exposure day 42.
